# Spontaneous neuronal avalanches as a correlate of access consciousness

**DOI:** 10.3389/fpsyg.2022.1008407

**Published:** 2022-10-21

**Authors:** Giovanni Rabuffo, Pierpaolo Sorrentino, Christophe Bernard, Viktor Jirsa

**Affiliations:** Institut de Neurosciences des Systemes, Aix-Marseille University, Marseille, France

**Keywords:** consciousness, neuronal avalanches, criticality, conscious access, resting state, spontaneous activity

## Abstract

Decades of research have advanced our understanding of the biophysical mechanisms underlying consciousness. However, an overarching framework bridging between models of consciousness and the large-scale organization of spontaneous brain activity is still missing. Based on the observation that spontaneous brain activity dynamically switches between epochs of segregation and large-scale integration of information, we hypothesize a brain-state dependence of conscious access, whereby the presence of either segregated or integrated states marks distinct modes of information processing. We first review influential works on the neuronal correlates of consciousness, spontaneous resting-state brain activity and dynamical system theory. Then, we propose a test experiment to validate our hypothesis that conscious access occurs in aperiodic cycles, alternating windows where new incoming information is collected but not experienced, to punctuated short-lived integration events, where conscious access to previously collected content occurs. In particular, we suggest that the integration events correspond to neuronal avalanches, which are collective bursts of neuronal activity ubiquitously observed in electrophysiological recordings. If confirmed, the proposed framework would link the physics of spontaneous cortical dynamics, to the concept of ignition within the global neuronal workspace theory, whereby conscious access manifest itself as a burst of neuronal activity.

## 1. Introduction

The origin of subjective lived experience, generally referred to as Consciousness, poses some of the most fascinating scientific and philosophical questions (Nagel, 1974). The advances of paradigms from the field of cognitive psychology, as well as theoretical models from computational neuroscience, have been pushing for refined definitions of Consciousness, and for a unified framework to understand and guide the interpretation of accumulating experimental evidences (Wiese, 2020; Melloni et al., 2021; Signorelli et al., 2021). A major distinction between “phenomenal” and “access” Consciousness was proposed (Block, 2005). Phenomenal (P-)Consciousness refers to raw experience (qualia; Chalmers, 1995). Access (A-)Consciousness refers to the availability of information for explicit reasoning and rational control. The latter definition allowed precise dissection of brain activity upon sensory stimuli that are accompanied by consciousness vs. stimuli that are not. In particular, task-based neuroimaging experiments led to the identification of robust neuronal correlates of A-Consciousness (NCC; Dehaene and Changeux, 2011; Aru et al., 2012). Generally speaking, event-related potentials (ERPs) display a subliminal early response in sensory areas regardless of the presence of Consciousness, then followed by characteristic waves of activity whose magnitude marks the presence or absence of perceptual awareness. For example, visual awareness negativity observed 200 ms after a visual stimulus, and enhanced P3 amplitude observed after 300 ms are often considered candidate NCCs (Koivisto and Revonsuo, 2010; Salti et al., 2012; Koivisto and Grassini, 2016). The Global Neuronal Workspace (GNW) hypothesis describes the crossing of the subliminal threshold and the consequent wave of activation as a “global ignition” (Mashour et al., 2020), which is postulated to be necessary for conscious access. These experiments elegantly identified the correlates of the conscious experience elicited by artificial stimuli. However, the required controlled experimental settings are far from naturalistic scenarios, where external and endogenous stimuli occur across multiple spatial and temporal scales. Another way of studying consciousness is by investigating the organizational principles of brain activity in altered states of consciousness. Studying functional imaging in such conditions led to the observation that the capability of supporting consciousness goes along with increasingly complex brain activities, which can be evaluated by using signal diversity measures such as Lempel-Ziv complexity (Casali et al., 2013; Schartner et al., 2015; Arena et al., 2021). In fact, the degree of complexity in brain signals provides a new mean to assess the presence of consciousness in a clinical setting, regardless of patient responsiveness (Sanders et al., 2012). The use of complexity measures has been influenced by the Integrated Information Theory (IIT; Oizumi et al., 2014; Tononi et al., 2016), which proposed the scalar quantity Φ as a measure of the quantity and quality of conscious experience, which concerns P-consciousness. In fact, it is possible to interpret Φ as a measure of information-processing complexity as well as dynamical systems complexity (Mediano et al., 2022). From a neurodynamical point of view, consciousness was associated to the brain being able to spontaneously explore a rich dynamical repertoire of network states, whereas unconscious states were associated to a less complex network dynamics (Uhrig et al., 2018; Demertzi et al., 2019). In fact, even at rest, when the brain is not involved in any specific task, large-scale brain activity dynamically organizes in communities of strongly correlated brain regions, or resting state networks, as observed by dynamic Functional Connectivity measures (Preti et al., 2017). Notably, resting state networks do not evolve continuously, nor periodically, but rather in aperiodic bursts of network co-fluctuations (Tagliazucchi et al., 2012; Zamani Esfahlani et al., 2020; Rabuffo et al., 2021). Similarly, large scale brain activity is often described as alternating segregated moments, in which regional activities are prominently independent from each other, to integrated moments when large-scale interaction occurs (Sporns, 2013; Deco et al., 2015; Shine, 2019).

While the importance of such dynamic reconfiguration for brain function is generally recognized (Lord et al., 2017), it is not clear how the spontaneous switching between segregated and integrated states relates to A- and P- Consciousness. For example, let us suppose that a visual stimulus is flashed to our retina. Does the presence of either brain state (i.e., segregated or integrated) affect the probability of such stimulus to gain conscious access? In this work we propose that information is collected predominantly during the segregated state, and that part of such information gains conscious access at the subsequent integrated state (see Section 4). In particular, we identify a fine-grained correlate of A-Consciousness corresponding to large-scale bursts of neuronal activations, interpreted as integration events. In the next chapter we associate these salient events to neuronal avalanches, as understood in the context of “brain criticality” theory. We review recent developments in Consciousness studies within this framework, we draw postulates of our hypotheses and propose experiments to test them.

## 2. Avalanches and consciousness

Consciousness should be understood within the physical principles governing the brain (Cosmelli et al., 2007; Werner, 2007). A widely discussed hypothesis is that, likewise other complex systems outside of equilibrium, the brain self regulates around a critical point i.e., at the edge of a second-order phase transition (Cocchi et al., 2017; OByrne and Jerbi, 2022), a property that is known as Self-Organized Criticality (SOC; Bak et al., 1987; Plenz et al., 2021). SOC offers an attractive theoretical framework for the brain, since it predicts the empirical evidences of optimal information processing (Shew et al., 2011; Shew and Plenz, 2013), dynamical range (Kinouchi and Copelli, 2006; Larremore et al., 2011), and maximization of metastability (Tognoli and Kelso, 2014). It was previously suggested that SOC could serve as a framework for Consciousness as well (Werner, 2009; Carhart-Harris et al., 2014; Tagliazucchi, 2017; Walter and Hinterberger, 2022). In fact, criticality was linked to IIT as a necessary condition for integration of information (Kim and Lee, 2019; Popiel et al., 2020), and it is compatible with predictions from the GNW hypothesis (Tagliazucchi, 2017), among other frameworks. The typical signature of SOC is the presence of neuronal avalanches, corresponding to sudden chains of neuronal activations across the brain. Neuronal avalanches are typically characterized by their duration—up to a few hundreds milliseconds—and their size i.e., the number of regions recruited. The distribution of the sizes of the avalanches follows a power-law, which indicates that these events span several orders of magnitude. In fact, neuronal avalanches can be consistently observed at the neuronal level using local multielectrode arrays (Beggs and Plenz, 2003), as well as at the whole-brain level using EEG (Palva et al., 2013), MEG (Shriki et al., 2013), SEEG (Priesemann et al., 2013), and fMRI (Tagliazucchi et al., 2012). It is to be noted that the outburst of an avalanche corresponds to the strong co-fluctuation of a set of brain regions, which promotes resting-state network dynamics (Tagliazucchi et al., 2012; Zamani Esfahlani et al., 2020; Rabuffo et al., 2021), among other signal properties. We propose that large neuronal avalanches support large-scale integration and are a candidate NCC at a fine-grained temporal scale. Accordingly, our hypotheses also apply in conditions such as resting-state. In our framework conscious access to external stimuli, would depend on the relative timing of the stimulus with respect to the spontaneous background avalanche dynamics.

## 3. Localize consciousness

An open question is the location of the physical substrate of a conscious experience. While it is generally accepted that Consciousness involves a distributed process across the brain regions, a major dichotomy lies in the anterior or posterior localization (Boly et al., 2017; Odegaard et al., 2017). For example, it was suggested that content-specific NCCs lay in neuronal ensembles within a hot zone situated in posterior cortical regions (Koch et al., 2016). Other evidences suggest that the prefrontal cortex is fundamental for the ignition upon (and therefore the conscious access to) visual inputs (Joglekar et al., 2018; Van Vugt et al., 2018). How these studies explain consciousness in absence of experimentally controlled stimuli is still open to debate (Mashour, 2018). Furthermore, evoked activation patterns underlying a conscious percept depend on the nature of the stimulus e.g., visual vs. auditory (Eriksson et al., 2007). Our proposal of linking A-Consciousness to spontaneous neuronal avalanches allows to interpret these results in the framework of resting-state activity. Functional imaging at rest revealed that healthy brains are characterized by a high-number of network reconfigurations (brain flexibility), a property which is disrupted in neurodegenerative diseases (Sorrentino et al., 2021a). In the context of dynamical systems theory, brain flexibility derives from the multi-stability of the dynamical repertoire (Golos et al., 2015), which has been linked to cognition (Kelso, 2012). Being dynamical in nature, avalanches recruit several modules in a flexible way, acting as a physical manifestation of multi-stability. We propose that fine-grained NCCs are coded into ever-changing patterns of network activations, which manifest as neuronal avalanches. However, we argue that a certain threshold size and/or topographic boundary should exist for an avalanche in order to be relevant for conscious access (see Section 5). Hence, the question of the localization of consciousness is reinterpreted from a network perspective, whereby the role that individual regions play into the spread and reconfiguration of neuronal avalanches is not homogeneous. In fact, the probability of cascading along a specific brain network resembles the structural connectome (Sorrentino et al., 2021b), suggesting that avalanches follow preferential pathways laid out by the neuronal projections. Thus, the topological role of a region within the connectome will partly define its relevance toward the spreading of perturbations on the large-scale. This is in line with the fact that the effect of a lesion on the large-scale dynamics correlate very poorly with the size of the lesion, while the topological role of the lesion carries much higher predictive power (Gratton et al., 2012).

## 4. Brain-state dependence of consciousness

**Figure 1:**
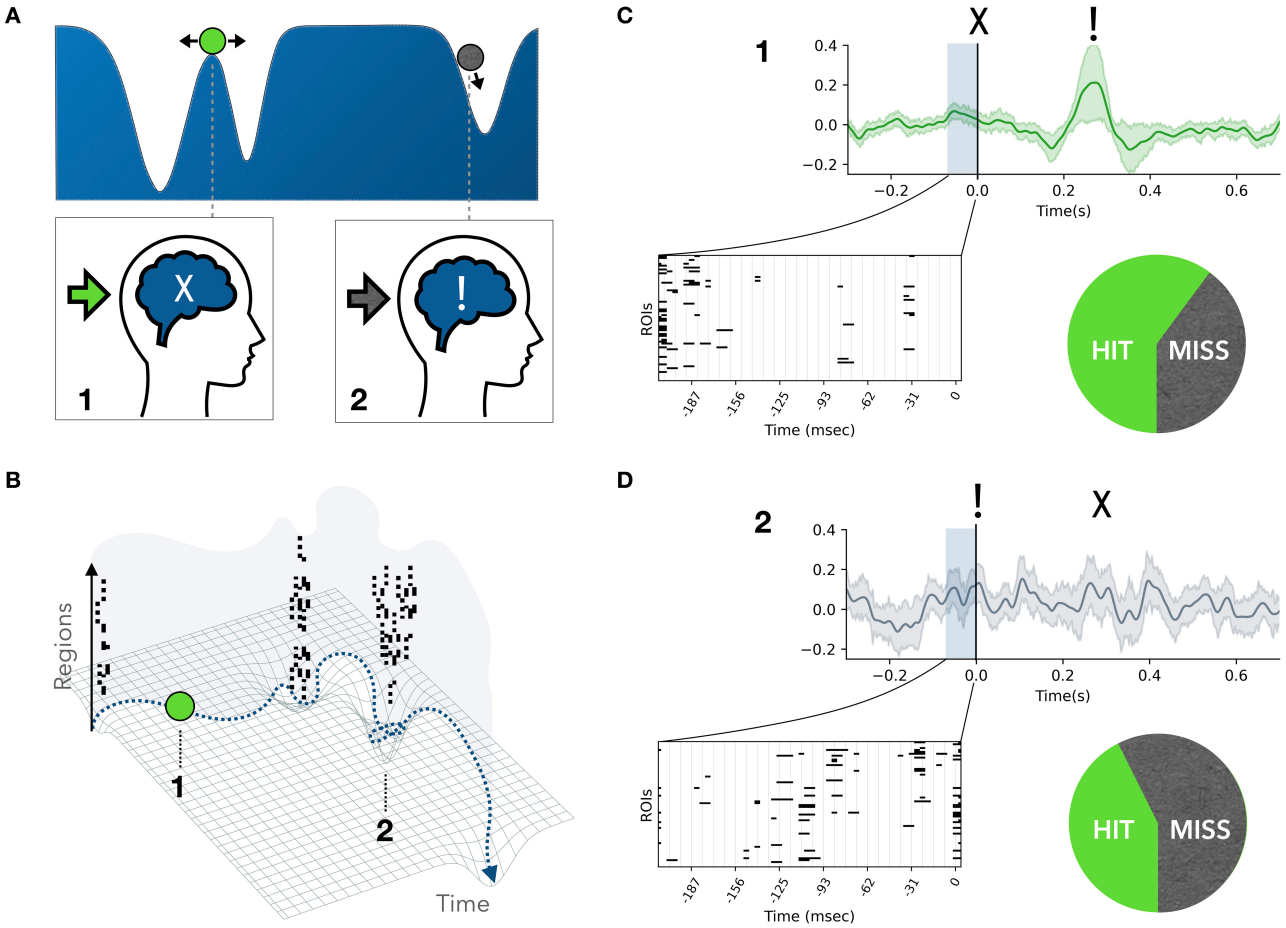
Conceptual framework. **(A,B)** The brain dynamics can be represented as a ball rolling on a landscape. During an inter-avalanche interval (state 1) the system explores the high plateau and can be easily pushed into a well by a perturbation. Therefore, in state 1 the system is maximally receptive to an incoming stimulus (green arrow), but not aware of it yet (marked by “X”). On the contrary, during an avalanche (state 2) the system is falling into a well, and an external perturbation has less impact on the system (gray arrow). An avalanche is characterized by the simultaneous fluctuation of several regions above a fixed threshold (marked by black bins). Therefore, state 2 supports the formation of a large-scale network of highly co-activated brain regions. We interpret this as a moment of integration of information across regions, where conscious access to previously collected information is achieved (marked as “!”). **(C,D)** Access to conscious processing occurs few hundred milliseconds after a stimulus is sent (at time “zero”), and this event is generally marked by a late wave of activations (ignition). Let us suppose that in an experimental setup a stimulus intensity is tuned such that perceptual awareness and the associated ignition occur in fifty percent of trials. We predict that if the stimulus is sent during an inter-avalanche interval **(C)**, the probability of gaining conscious access a few hundred milliseconds later is greater than fifty percent. Accordingly, the probability drops below fifty percent if the stimulus coincides with a large avalanche **(D)**.

A recent study on monkeys suggested that conscious access is state-dependent, whereby fluctuations in the behavioral and neurophysiological markers before the visual stimulus were related to variations in stimulus detection (Van Vugt et al., 2018). Similarly, we expect a brain-state dependence of conscious access, given that the presence or absence of an avalanche mark qualitatively distinct states in the functional evolution of brain dynamics. In fact, neuronal ensembles are not always in communication, and windows of coherence are expected to be necessary for information routing (Fries, 2005). In particular, large-scale coordination displays the dynamic alternation of regional segregation and integration (Friston, 2009; Sporns, 2013; Deco et al., 2015). The sequential recruitment of neuronal ensembles during avalanches elicits characteristic brain networks, and corresponds to periods of increased functional connectivity. Thus, under the communication through coherence hypothesis (Fries, 2005), we assume that neuronal avalanches underlie periods of integration. As a first conceptualization of our framework, based on the idea that integration of information is a prerequisite for consciousness (Tononi et al., 1998a), we posit that avalanches spreading across the brain correspond to discrete conscious access events, where previously acquired information is broadcast in the brain. On the one hand, we argue that avalanche states are poor receptive states, since the already-entrained populations are generally less susceptible to external stimuli. On the other hand, if a stimulus is received during an inter-avalanche interval, it has a higher probability of triggering a new avalanche and of gaining conscious access after a few hundreds milliseconds. We can express these concepts in the language of dynamical systems and manifolds. In this representation, the state of the system can be thought of as a ball rolling over a surface (manifold), whose wells (attractors) represent quasi-stable configurations of the system (Figures 1A,B). The brain activity spends a large amount of time in one attractor, and then quickly transitions to the next one. Over time, multiple attractors (i.e., “the landscape”) will have been visited. Hence, rather than stationary, the patterns of activity in the data will be *metastable* (Haken, 1991; Friston, 1997; Huys et al., 2014; Deco and Kringelbach, 2016; Roberts et al., 2019; Shine et al., 2019).

Neuronal avalanches correspond on the manifold to moments in which the ball is (falling) into a well (and, as said, conscious access to previously collected stimuli is gained). In this situation it is harder for a new incoming external perturbation (stimulus) to change the trajectory of the system. On the contrary, when the ball is on the peak, the system is more susceptible to external stimuli, which can easily push the ball into a new attractor (i.e., update the conscious percept). Summarizing, we suggest that the brain alternates receptive windows (inter-avalanche intervals), where incoming information is collected, to punctuated short-lived integration events (large avalanches), corresponding to conscious access to the previously collected information.

Finally, we propose a general experimental setting to test these hypotheses. In task-based experiments it is possible to fine-tune the duration and intensity of a sensory stimulus (e.g., visual) to a target subject-specific probability of such stimulus gaining conscious access. Our hypotheses predict an increased probability of missing the target if that is presented during a large avalanche. This can be tested by simultaneously recording the spontaneous avalanche state at the moment of the presentation of the stimulus (Figures 1C,D). However, given the aperiodic occurrence of avalanches, it is challenging to time the presentation of a stimulus to a specific brain state. To overcome the problem, we propose to exploit an important property of neuronal avalanches, namely the shape collapse (Sethna et al., 2001). When an avalanche breaks out, regions are gradually recruited until a maximum is reached, and then the activity fades away equally gradually. This phenomenon holds across orders of magnitude, which allows all avalanche profiles to be collapsed into a parabolic shape by a fixed scaling exponent, a property expected theoretically for a class of universality of critical phenomena (Papanikolaou et al., 2011; Laurson et al., 2013; Miller et al., 2019). This implies a short-term form of predictability over the course of an avalanche. In fact, once an avalanche reaches the maximum recruitment after N steps, one can predict that it will persist for the next N time steps, symmetrically collapsing in time, up to few hundred milliseconds for the longest avalanches. This would provide a time-window to synchronize the stimulus to the background avalanche state. Importantly, such probability can be evaluated in the absence of any task or behavior using a no-report paradigm, such as Sergent et al. (2021).

## 5. Discussion

The brain activity is functionally dynamic and conscious processes might depend on the spontaneous alternation of integrated and segregated functional states. Neuronal avalanches are ubiquitously observed in brain imaging recordings, in association with expected features underpinning Consciousness, such as complexity, flexibility and multistability. In this work, we propose an overarching framework to link a number of empirical findings related to consciousness and its neuronal correlates. Our hypotheses remain highly hypothetical. However, we propose an experimental design to test our framework. In detail, we propose that a computational cycle for consciousness consists of a subliminal phase, where the brain operates in a segregated state and external stimuli are gathered, and an integrated state manifesting as a neuronal avalanche, where the previously acquired information can gain conscious access. In our framework, conscious access manifest itself as large-scale bursts of activation. Such bursts can be induced by external stimuli (e.g., ignition), but are also empirically present in spontaneous resting-state activity (e.g., neuronal avalanches). Hence, our hypotheses ought to explain Consciousness also in absence of a clear task e.g., when we are mind-wandering. In general, in order to identify the necessary and sufficient conditions for an avalanche to support Consciousness, it is important to distinguish between neuronal avalanches generated during conscious or unconscious states. In this context, the topography of the networks recruited by an avalanche is expected to be relevant. In fact, specific neuronal circuits supporting the emergence and fading of Consciousness have been identified in the literature. As an example, the cortico-cortical and thalamo-cortical loops are associated to both states and contents of Consciousness (Dynamic Core hypothesis; Edelman and Tononi, 2000). At the mesoscale, the pyramidal neurons in layer 5 (L5p) in the cortex have been identified as a key relay between the cortico-cortical and the thalamo-cortical loops. As such, the involvement of L5p has been hypothesized as a necessary condition for cortical processes to support consciousness (Aru et al., 2019). Recent evidence highlight changes in L5p activity during anesthesia, such as increased low-frequency power (Bastos et al., 2021), and selective synchronization (Bharioke et al., 2022). Supposedly, these changes impair the dendritic-to-soma coupling (Aru et al., 2020; Suzuki and Larkum, 2020), thereby disconnecting the thalamo-cortical broadcasting system which would no longer support consciousness. In the light of this evidence, we hypothesize that a conscious state should be supported by avalanches that contribute to the integration of these systems, which is more likely for large-sized avalanches. However, we do not exclude that access consciousness can be supported by small avalanches, provided that they recruit the relevant structures. Classical statistical measures related to neuronal avalanches (e.g., power law distribution, branching ratio etc..), often used as a signature of (or against) criticality, might not be optimal to assess conscious vs. unconscious states as they disregard both the topography and the temporal organization of brain activity. The temporal factor is also crucial to the emerge of consciousness, as a large body of research suggests that Consciousness involves multiple characteristic timescales, spanning from the perceptive threshold ( 15 − 50*ms*) to the extension ( 100 − 500*ms*) and the retention ( 3 − 7*s*) of content (see Singhal and Srinivasan, 2021 and references within). While a number of frameworks describing the temporal hierarchy of conscious perception have been proposed (e.g., Pöppel, 1997; Varela, 1999; Poppel, 2004; Wanja, 2017), a clear biophysical mechanism encompassing fast, intermediate and slow temporal scales is still missing. To this regard, neuronal avalanches offer a promising overarching framework to explain the spontaneous emergence of this hierarchy of timescales. In fact, the duration of neuronal avalanches is rather short on average (tens of milliseconds; comparable to the perceptive threshold) with respect to the duration of IAI (hundreds of milliseconds; which is closer to the extensional timescales). Furthermore, it was shown that the dynamics of avalanches possesses a slower timescale in the order of a few seconds (Rabuffo et al., 2021), which is comparable to the retentional timescale. However, it must be noted that a separation of timescales predicted by the SOC framework is not clearly observed in experimental recordings (Lombardi et al., 2012, 2021; Priesemann et al., 2014). It was previously shown that large avalanches tend to couple with longer preceding and following IAI (Lombardi et al., 2016), which might be related to the proposed hypothesis of information gathering during IAI and conscious access during the following large avalanches (and also be of use to design the experiments). It had been proposed that cognition and perception might operate in discrete cycles (VanRullen and Koch, 2003; Madl et al., 2011; VanRullen, 2016). The identification of these cycles with recurrent neuronal avalanches suggests that fast cognitive processes might be aperiodic, and that the duration and intensity of a percept could be modulated in time (Herzog et al., 2016), likewise the duration and size of neuronal avalanches.

Arguably, one of the most used ontologies for the description of neuronal activity in neuroscience refers to neuronal oscillations (Buzsaki, 2006). In this framework, transient neuronal synchronization events are considered as a correlate of high-order cognitive processing (Tononi et al., 1998a,b; Rodriguez et al., 1999; Srinivasan et al., 1999; Engel and Singer, 2001; Ward, 2003; Melloni et al., 2007). In particular, synchronization of neuronal populations has been proposed to mediate the merging of multiple local processes into a single conscious experience (Singer, 2001). Hence, a mechanistic framework for consciousness should ideally portray both oscillations and avalanches. Recent works has provided both theoretical and experimental evidence in this direction, showing nested oscillations coexisting with neuronal avalanches (Gireesh and Plenz, 2008). In the same vein, it was shown *in-silico* that neuronal avalanches occur at a critical (asynchronous-to-synchronous) phase transition, where they coexist with incipient oscillations (Di Santo et al., 2018). However, while SOC provides a candidate framework for a unified theory of Consciousness (Melloni et al., 2021), a critical state is not strictly required for explaining the presence of spontaneous scale-free neuronal avalanches (e.g., Buend́ıa et al., 2020a, 2022). For example, it was suggested that during deep sleep or under anesthesia, the brain self-organizes at the edge of bistability (SOB, a first order phase transition Buend́ıa et al., 2020b), rather than SOC (Priesemann et al., 2013). Furthermore, other bursting phenomena such as EEG micro states (Britz et al., 2009; Michel and Koenig, 2018) and neuronal assemblies (Papadimitriou et al., 2020) have been linked to cognition and perception. Notably, the global ignition proposed by the GNW hypothesis and observed when an external stimulus gains conscious access, might be understood as an avalanche of neuronal activations.

In conclusion, in this manuscript we propose a unifying framework to link both spontaneous and induced conscious access to neuronal avalanches. Neuronal avalanches are a solid finding in large-scale human recordings, which allow to state testable hypotheses and design the experiments accordingly. The presence of ignition when a stimulus gains conscious access, as predicted by the GNW hypothesis, and the loss of complexity in unconscious states, predicted by IIT, can both be understood within the proposed framework. Importantly, neuronal avalanches also offer a mathematically solid approach to link the properties observed on the large-scale to hypothetical microscopic mechanisms, which might offer a window to approach consciousness in mechanistic terms.

## Data availability statement

The original contributions presented in the study are included in the article/supplementary material, further inquiries can be directed to the corresponding author.

## Author contributions

GR and PS conceived the presented ideas and contributed to the writing of the manuscript. GR designed the figure. CB and VJ supervised the findings and the writing of this work. All authors discussed and contributed to the final manuscript.

## Funding

This work was supported by the grant ANR-17-CE37-0001-CONNECTOME, by the European Union's Horizon 2020 research and innovation programme under grant agreement No. 945539 (SGA3) Human Brain Project and VirtualBrainCloud No. 826421.

## Conflict of interest

The authors declare that the research was conducted in the absence of any commercial or financial relationships that could be construed as a potential conflict of interest.

## Publisher's note

All claims expressed in this article are solely those of the authors and do not necessarily represent those of their affiliated organizations, or those of the publisher, the editors and the reviewers. Any product that may be evaluated in this article, or claim that may be made by its manufacturer, is not guaranteed or endorsed by the publisher.
